# A Genomic Bayesian Multi-trait and Multi-environment Model

**DOI:** 10.1534/g3.116.032359

**Published:** 2016-06-24

**Authors:** Osval A. Montesinos-López, Abelardo Montesinos-López, José Crossa, Fernando H. Toledo, Oscar Pérez-Hernández, Kent M. Eskridge, Jessica Rutkoski

**Affiliations:** * Biometrics and Statistics Unit and Global Wheat Program of the International Maize and Wheat Improvement Center (CIMMYT), México, D.F. 06600, México; † Departamento de Estadística, Centro de Investigación en Matemáticas (CIMAT), Guanajuato, Guanajuato 36240, México; ‡ Department of Biology and Agriculture, University of Central Missouri, Warrensburg, Missouri 64093; § Statistics Department, University of Nebraska, Lincoln, Nebraska, 68583

**Keywords:** multi-trait, multi-environment, Bayesian estimation, genome-enabled prediction, genomic selection, GenPred, shared data resource

## Abstract

When information on multiple genotypes evaluated in multiple environments is recorded, a multi-environment single trait model for assessing genotype × environment interaction (G × E) is usually employed. Comprehensive models that simultaneously take into account the correlated traits and trait × genotype × environment interaction (T × G × E) are lacking. In this research, we propose a Bayesian model for analyzing multiple traits and multiple environments for whole-genome prediction (WGP) model. For this model, we used Half-t priors on each standard deviation term and uniform priors on each correlation of the covariance matrix. These priors were not informative and led to posterior inferences that were insensitive to the choice of hyper-parameters. We also developed a computationally efficient Markov Chain Monte Carlo (MCMC) under the above priors, which allowed us to obtain all required full conditional distributions of the parameters leading to an exact Gibbs sampling for the posterior distribution. We used two real data sets to implement and evaluate the proposed Bayesian method and found that when the correlation between traits was high (>0.5), the proposed model (with unstructured variance–covariance) improved prediction accuracy compared to the model with diagonal and standard variance–covariance structures. The R-software package Bayesian Multi-Trait and Multi-Environment (BMTME) offers optimized C++ routines to efficiently perform the analyses.

Since the whole-genome prediction (WGP) model of Meuwissen *et al.* (2001), practical results have shown that genomic selection (GS) using Bayesian and non-Bayesian linear regression models improves prediction accuracy compared to conventional and pedigree selection (de los Campos *et al.* 2009, 2010; Crossa *et al.* 2010, 2011; Heslot *et al.* 2012; Pérez-Rodríguez *et al.* 2012). With GS, genomic breeding values are estimated as the sum of marker effects for genotyped individuals in the testing or prediction population. The marker effects are estimated simultaneously using a training population that contains phenotyped and genotyped individuals.

In plant breeding, most of the available methods for WGP are useful for analyzing a single trait measured either in a single environment or in multi-environments with the incorporation of genotype × environment interaction (G × E) (Burgueño *et al.* 2012; Heslot *et al.* 2014; Jarquín *et al.* 2014, Montesinos-López *et al.* 2015, López-Cruz *et al.* 2015). However, researchers often face situations in which multiple traits are measured across multiple environments. For example, crop breeders record phenotypic data for multiple traits such as grain yield and its components (*e.g.*, grain type, grain weight, biomass, *etc*.), grain quality (*e.g.*, taste, shape, color, nutrient content), and tolerance to biotic and abiotic stresses. They often aim to improve all these multiple correlated traits simultaneously or to predict the ones that are difficult to measure with those that are easy to measure. However, it is common practice to perform an independent analysis and genomic prediction on a single phenotypic trait.

The advantage of jointly modeling multiple traits compared to analyzing each trait separately is that the inference process appropriately accounts for the correlation among the traits, which helps to increase prediction accuracy, statistical power, parameter estimation accuracy, and reduce trait selection bias (Henderson and Quaas 1976; Pollak *et al.* 1984; Schaeffer 1984). In the context of WGP, Jia and Jannink (2012), Guo *et al.* (2014), and Jiang *et al.* (2015) found that joint prediction of multiple traits benefits from genetic correlation between traits and significantly improves prediction accuracy compared to single trait methods, specifically for low-heritability traits that are genetically correlated with a high-heritability trait. Jia and Jannink (2012) also found better prediction accuracy for multiple traits than for single traits when phenotypes are not available for all individuals and traits. Therefore, there is evidence that multiple trait analysis is useful to predict yet-to-be observed phenotypes in plant and animal breeding when selecting unphenotyped candidates early through the prediction of their genomic breeding values. Multi-trait analysis has also been found to substantially increase prediction accuracy when some traits are observed in all individuals but the trait of interest is not observed in the individuals in the test set (Pszczola *et al.* 2013; Rutkoski *et al.* 2016).

Multivariate analysis of continuous outcomes is well established in statistical literature (Johnson and Wichern 1992). However, the available methods cannot be applied in a straightforward manner for WGP, since the number of independent variables (*p*) is usually larger than the available sample size (*n*). The genomic best linear unbiased predictor (GBLUP) WGP model can be implemented in standard software for multiple traits and multiple environments by taking into account two-way interaction terms and estimating separable unstructured covariance matrices of the form A⊗B (where A and B are the corresponding covariance matrices of factors A and B, respectively). However, these software programs are unable to estimate separable unstructured variance–covariance matrices of the form A⊗B⊗C for three-way interaction terms. For this reason, in this situation, at least one of the variance–covariance components is assumed to be identity or a new variable is created by merging two factors and estimating a covariance matrix with only two components as $A⊗B^{*}$, where $B^{*}$ contains the variance–covariance of two factors, but each component cannot be separated. Also, univariate Bayesian inference has been proposed and extensively implemented in WGP models (Gianola 2013). The Bayesian alphabet methods (Bayes A, Bayes C, and Bayes Cπ) have been extended for multiple trait analysis (de los Campos and Gianola 2007; Calus and Veerkamp 2011; Jia and Jannink 2012; Guo *et al.* 2014) and most recently, Jiang *et al.* (2015) proposed a Bayesian multivariate antedependence model.

Despite evidence of the increased prediction accuracy of WGP models incorporating G × E (Burgueño *et al.* 2012; Jarquín *et al.* 2014, Montesinos-López *et al.* 2015; López-Cruz *et al.* 2015) and of WGP models for multi-trait data, statistical models for analyzing continuous data for simultaneously assessing multi-traits and multi-environments are lacking. Thus, the integration of these two approaches in one unified WGP model is required (Jiang *et al.* 2015). This unified WGP model would be useful in two cases: (i) when individuals are measured for all traits in one environment, but only some traits in other environments; and (ii) when some traits are recorded in only a subset of individuals in all environments. This model would be useful not only in plant breeding but also in animal breeding, where genetic evaluation of many traits is performed on a weekly basis by many breeding programs globally. It is also possible to integrate other advantageous strategies such as the antedependence model to incorporate dominant and epistatic effects.

All the Bayesian methods developed so far for multiple trait analysis use the Inverse-Wishart (IW) conjugate family of distributions as priors for the covariance matrices between traits. However, Gelman (2006) and Huang and Wand (2013) argued against using IW priors for covariance matrices because they impose a degree of informativity and the posterior inferences are sensitive to the choice of hyper-parameters. Recently, Huang and Wand (2013) proposed a scale mixture approach involving an IW distribution and independent Inverse-Gamma (IG) distributions for each dimension as priors for the covariance matrix parameters. The ensuing covariance matrix distribution is such that all standard deviation parameters have Half-t distributions and the correlation parameters have uniform distributions on (−1,1) for a particular choice of the IW shape parameter. The advantage of this approach is that it is possible to choose shape and scale parameters that achieve arbitrary high noninformativity of all standard deviations and correlation parameters (Huang and Wand 2013). However, the model proposed by Huang and Wand (2013) is a standard mixed model with correlated errors that does not include interaction terms of any kind and does not consider three-way interaction.

In this study, we propose a Bayesian method that integrates the analysis of multi-traits and multi-environments and takes into account trait × genotype × environment interaction (T × G × E) in a unified WGP model. We used Half-t priors on each standard deviation term and uniform priors on each correlation to achieve high noninformativity and posterior inferences that are not sensitive to the choice of hyper-parameters. We illustrate the use of the unified Bayesian Multi-Trait and Multi-Environment (BMTME) method in simulated data sets and two real data sets (one maize and one wheat) including multiple traits measured on wheat and maize lines evaluated in multiple environments and genotyped with dense molecular markers. We also provide an R package called BMTME that can be used to fit the proposed methods.

## Methods

### Statistical model

We use $y_{ijk}^{\left(l\right)}$ to represent the normal response from the k th replication of the j th line in the i th environment for the l th trait (i=1,2,…,I, j=1,2,…,J, k=1,2,…,K, l=1,…,L), where K represents the number of replicates of each line in each environment and L denotes the number of traits under study. To present the theory in a simple manner, we will use I=3 and L=3. Therefore, the total number of observations for the l th trait is n=I×J×K. We propose the following linear mixed model for each trait:

$$y_{ijk}^{\left(l\right)}=E_{i}^{\left(l\right)}+g_{j}^{\left(l\right)}+gE_{ij}^{\left(l\right)}+e_{ijk}^{\left(l\right)}$$ (1)

where $E_{i}^{\left(l\right)}$ represents the i th environment for the l th trait and is assumed as a fixed effect, $g_{j}^{\left(l\right)}$ represents the genomic effect of j th line in the l th trait and is assumed as random effect, $gE_{ij}^{\left(l\right)}$ is the interaction between the genomic effect of the j th line and the i th environment for the l th trait and is assumed a random effect, and $e_{ijk}^{\left(l\right)}$ is a random error term associated with the k th replication of the j th line in the i th environment for the l th trait. To take into account the correlation between traits, one could use the following L variate linear mixed model:

$$y_{ijk}=X_{ijk}\;\beta +Z_{1ijk}b_{1j}+Z_{2ijk}b_{2ij}+e_{ijk}$$ (2)

$y_{ijk}={\left[y_{ijk}^{\left(1\right)},\ldots ,y_{ijk}^{\left(3\right)}\right]}^{T}$, $X_{ijk}=\left[\begin{array}{ccc} x_{i}^{T(1)} & 0 & 0\\ 0 & x_{i}^{T(2)} & 0\\ 0 & 0 & x_{i}^{T(3)} \end{array}\right],\beta ={\left[\beta^{T\left(1\right)},\beta^{T\left(2\right)},\beta^{T\left(3\right)}\right]}^{T},$ $Z_{1ijk}=Z_{2ijk}=I_{3},$ $b_{1j}={\left[b_{1j}^{\left(1\right)},\ldots ,b_{1j}^{\left(3\right)}\right]}^{T}∼N_{L}\left(0,\Sigma_{t}\right),\Sigma_{t}$ is the genetic covariance matrix between traits and is assumed unstructured, $b_{2ij}={\left[b_{2ij}^{\left(1\right)},\ldots ,b_{2ij}^{\left(3\right)}\right]}^{T},$ $e_{ijk}={\left[e_{ijk}^{\left(1\right)},\ldots ,e_{ijk}^{\left(3\right)}\right]}^{T}∼N_{L}\left(0,R_{e}\right)$, $R_{e}$ is the residual covariance matrix between traits and is assumed unstructured. $x_{i}^{T(l)}=\left[x_{i1}^{\left(l\right)},x_{i2}^{\left(l\right)},x_{i3}^{\left(l\right)}\right],$ $x_{ir}^{\left(l\right)}=1$ if the environment i is observed and 0 otherwise for the l th trait, for r=1,2,3; and l=1,2,3. $\beta^{T(l)}=\left[\beta_{1}^{\left(l\right)},\beta_{2}^{\left(l\right)},\beta_{3}^{\left(l\right)}\right],$ $x_{i}^{T(l)}\beta^{\left(l\right)}=E_{i}^{\left(l\right)},$ $b_{1j}^{\left(l\right)}=g_{j}^{\left(l\right)}$ and $b_{2ij}^{\left(l\right)}=gE_{ij}^{\left(l\right)}$. With model (1), we can perform a separate analysis for each trait, with the inconvenience that independence between the L traits is assumed. Model (2) can take into account and exploit the correlation between traits.

In matrix notation, the model given in equation (2) including all the information is expressed as:

$$Y=\mathrm{X\beta}+Z_{1}b_{1}+Z_{2}b_{2}+e$$ (3)

where Y is of order Ln×1, X is of order Ln×IL, β is of order IL×1, $Z_{1}$ is of order Ln×LJ, $b_{1}$ is of order LJ×1, $Z_{2}$ is of order Ln×IJL, $b_{2}$ is of order IJL×1_,_ and e is of order Ln×1. Then $b_{1}∼N\left(0,G_{1}\right),$ $b_{2}∼N\left(0,G_{2}\right)$ and e∼N(0,R), where $G_{1}=G_{g}⊗\Sigma_{t}$, ⊗ denotes a Kronecker product, $G_{2}=\Sigma_{E}⊗G_{1},$ where $\Sigma_{E}$ is assumed a diagonal matrix of order I×I, which indicates that we are assuming independence between environments. It is important to point out that the trait × environment (T × E) interaction term is included in the fixed effect β, while the trait × genotype (T × G) interaction term is included in the random effect $b_{1}$ and the three-way (T × G × E) interaction term is included in $b_{2}$. The errors are assumed to be correlated with the covariance defined as $R=I_{n}⊗R_{e}$. More flexible variance–covariances as diagonal or identity are straightforward. Also note that $G_{g}$ is of order J×J; therefore, $G_{1}$ is of order JL×JL and $G_{2}$ is of order IJL×IJL. The matrix of the genomic relationship between lines $G_{g}$, also known as Genomic Relationship Matrix (GRM), was calculated using the method of VanRaden (2008).

### Joint posterior density and prior specification

In this section, we provide the joint posterior density and prior specification for the Bayesian WGP Multiple Trait and Multiple Environment (BMTME) model. The joint posterior density of the parameter vector becomes:

$$P(\beta ,b_{1},b_{2},\Sigma_{t},\Sigma_{E},\sigma_{\beta }^{2},R_{e},a_{\beta },a,a_{E},a_{e})\propto P(Y|\beta ,b_{1},b_{2},R_{e})P(\beta |\sigma_{\beta }^{2})P(\sigma_{\beta }^{2}|a_{\beta })P(a_{\beta })P(b_{1}|\Sigma_{t})P(\Sigma_{t}|a_{1,}\ldots ,a_{L})P(a_{1,}\ldots ,a_{L})\times P(b_{2}|\Sigma_{t},\Sigma_{E})P(\Sigma_{E}|a_{E1,}\ldots ,a_{EI})P(a_{E1,}\ldots ,a_{EI})\times P(R_{e}|a_{e1,}\ldots ,a_{eL})P(a_{e1,}\ldots ,a_{eL})$$ (4)

where $a=\left(a_{1},\ldots ,a_{L}\right),a_{E}=\left(a_{E1},\ldots ,a_{EI}\right),a_{e}=\left(a_{e1},\ldots ,a_{eL}\right)$.

The notation Ω∼ Inverse-Wishart (κ,B) indicates that the density function of Ω is $\text{P}\left(\Omega \right)\propto {\left|B\right|}^{\frac{\kappa }{2}}{\left|\Omega \right|}^{-\frac{\kappa +p+1}{2}}\exp\left[-\frac{1}{2}tr\left(B\Omega^{-1}\right)\right],$ κ>0,B,Ω both are positive definite matrices. We assume that $\beta |\sigma_{\beta }^{2}∼N_{\mathrm{IL}}\left(\beta_{0},\Sigma_{0}\sigma_{\beta }^{2}\right),$ $\sigma_{\beta }^{2}|a_{\beta }∼IW\left(\nu_{\beta },2\nu_{\beta }/a_{\beta }\right)\text{ where }IW\left(\nu_{\beta },2\nu_{\beta }/a_{\beta }\right)$ denotes an Inverse-Wishart distribution with shape $\nu_{\beta }$ and scale parameters $2\nu_{\beta }/a_{\beta }$ with $a_{\beta }∼IG\left(\frac{1}{2},1/A_{\beta }^{2}\right)$, $\text{where }IG\left(\frac{1}{2},1/A_{\beta }^{2}\right)$ denote an IG distribution with shape 1/2 and scale parameters $1/A_{\beta }^{2}$. $b_{1}|\Sigma_{t}∼N_{JL}\left(0,G_{1}\right)$, $\Sigma_{t}|a_{1,}\ldots ,a_{L}∼IW\left(\nu_{t}+L-1,2\nu_{t}\text{diag}(\frac{1}{a_{1}},\ldots \frac{1}{a_{L}})\right)$, $a_{l}∼IG\left(\frac{1}{2},1/A_{l}^{2}\right)$ for l=1,…,L. $b_{2}|\Sigma_{t},\Sigma_{E}∼N_{IJL}\left(0,G_{2}\right)$, $R_{e}|a_{e1,}\ldots ,a_{eL}∼$$IW\left(\nu_{e}+L-1,2\nu_{e}\text{diag}\left(\frac{1}{ae_{1}},\ldots \frac{1}{ae_{L}}\right)\right)$ and $a_{el}∼IG\left(\frac{1}{2},1/A_{el}^{2}\right)$. Since $\Sigma_{E}=\text{diag}(\sigma_{E1}^{2},\ldots ,\sigma_{EI}^{2})$, the prior for $\sigma_{Ei}^{2}|a_{Ei}∼IW\left(\nu_{Ei},2\nu_{Ei}/a_{Ei}\right)$ with the prior for $a_{Ei}∼IG\left(\frac{1}{2},1/A_{Ei}^{2}\right)$ for i=1,…,I.

Next we combine the joint posterior density of the parameter vector (4) with the priors to obtain the full conditional distribution for parameters β, $\sigma_{\beta }^{2}$, $a_{\beta }$, $b_{1}$, $b_{2}$, $\Sigma_{t},$ $a,R_{e},a_{e}$. All full conditionals, as well as details of their derivations, are given in Appendix A.

### Gibbs sampler

In order to produce posterior means for all relevant model parameters, below we outline the exact Gibbs sampler procedure that we propose for estimating the parameters of interest. As is the case with Markov Chain Monte Carlo (MCMC) techniques, the ordering of draws is somewhat arbitrary; however, we suggest the following order:

Step 1. Simulate β according to the normal distribution given in Appendix A (A.1).
Step 2. Simulate $\sigma_{\beta }^{2}$ according to the IW distribution given in Appendix A (A.2).
Step 3. Simulate $a_{\beta }$ according to the IG distribution given in Appendix A (A.3).
Step 4. Simulate $b_{h}$ for h=1,2, according to the normal distribution given in Appendix A (A.4 and A.5).
Step 5. Simulate $\Sigma_{t}$ according to the IW distribution given in Appendix A (A.6).
Step 6. Simulate $a_{l},\text{for}\;l=1,2,\ldots ,L,$ according to the IG distribution given in Appendix A (A.7).
Step 7. Simulate $\sigma_{Ei}^{2},\text{for}\;i=1,\ldots ,I,$ according to the IW distribution given in Appendix A (A.8).
Step 8. Simulate $a_{Ei},\text{for}\;i=1,\ldots ,I,$ according to the IG distribution given in Appendix A (A.9).
Step 9. Simulate $R_{e}$ according to the IW distribution given in Appendix A (A.10).
Step 10. Simulate $a_{el},\text{for}\;l=1,2,\ldots ,L,$ according to the IG distribution given in Appendix (A.11).
Step 11. Return to step 1 or terminate when chain length is adequate to meet convergence diagnostics.

### Model implementation

The Gibbs sampler described above for the BMTME model was implemented as an R-software package. We performed a total of 60,000 iterations; 30,000 samples were used for inference because the first 30,000 were used as burn-in to decrease the MCMC errors in prediction accuracy. We did not apply thinning of the chains following the suggestions of Geyer (1992), MacEachern and Berliner (1994), and Link and Eaton (2012), who provide justification of the ban on subsampling MCMC output for approximating simple features of the target distribution (*e.g.*, means, variances, and percentiles).

We implemented the prior specification given in the previous section where the BMTME model was defined. The hyper-parameters used were for $\beta |\sigma_{\beta }^{2}∼N_{IL}\left(\beta_{0}=0_{IL}^{T},I_{IL}\times 10,000\right),$ for $\sigma_{\beta }^{2}|a_{\beta }$ we used $\nu_{\beta }=2,$ for $a_{\beta }$ we used $A_{\beta }=100,000,$ for $b_{1}|\Sigma_{t}∼N_{JL}\left(0,G_{1}\right)$, for $\Sigma_{t}|a_{1,}\ldots ,a_{L}$ we used $\nu_{t}=2$, for $a_{l}$ we used $A_{l}=100,000,$ for l=1,2,…,L, for $b_{2}|\Sigma_{t},\Sigma_{E}∼N_{IJL}\left(0,G_{2}\right)$, for $\sigma_{Ei}^{2}|a_{Ei}$ we used $\nu_{Ei}=2$ for $a_{Ei}$ we used $A_{Ei}=100,000$ for $i=1,2,\ldots ,I,\text{for}\;R_{e}|a_{e1,}\ldots ,a_{eL}\text{ we used}\nu_{e}=2,\text{for}a_{el}\text{we used}A_{el}=100,000\text{for }l=1,..,L$. All these hyper-parameters were chosen to lead weakly informative priors.

### Assessing prediction accuracy

We used two cross-validation schemes for generating training and validation sets that mimic two real situations a breeder might face. Cross-validation 1 (CV1) mimics a situation where lines were evaluated in some environments for all traits but some lines are missing in other environments; this is similar to cross-validation 2 of Burgueño *et al.* (2012). The other cross-validation scheme is CV2, which mimics a situation where a trait is lacking in all lines in one environment but present in the remaining environments (see Table D1 in Appendix D). In this case, information from relatives is used, and prediction assessment can benefit from borrowing information between lines across environments, and among correlated traits.

We implemented a 10-fold cross-validation with 80% of the observations in the training set and 20% in the testing set. Of the variety of methods for comparing the predictive posterior distribution to the observed data (generally termed “posterior predictive checks”), we used two criteria: the mean square error of prediction (MSEP) and the Pearson correlation. Models with small MSEP indicate better predictions, and higher correlation values indicate better predictions. The predicted observations were calculated with S collected Gibbs samplers as: ${\hat{Y}}^{(s)}=X\beta^{\left(s\right)}+Z_{1}b_{1}^{\left(s\right)}+Z_{2}b_{2}^{\left(s\right)}$, where $\beta^{\left(s\right)},b_{1}^{\left(s\right)},\text{and}\;b_{2}^{\left(s\right)}$ are estimates of β, $b_{1}$, and $b_{2}$ in the s*th* collected sample.

### Simulation data

To illustrate the parameter estimation of the proposed BMTME method, a small simulation experiment was conducted. The data were simulated based on model (3) with three environments, three traits, 80 genotypes, and 20 replications. We assumed that $\beta^{T}=[$15,8,7,12,6,7,14,9,8], where the first three β coefficients belong to traits 1, 2, and 3 in environment 1, the second three values for the three traits in environment 2 and the last three for environment 3,$\Sigma_{t}=\left[\begin{array}{ccc} 0.600 & 0.466 & 0.551\\ 0.466 & 0.500 & 0.503\\ 0.551 & 0.503 & 0.700 \end{array}\right]$, $R_{e}=\left[\begin{array}{ccc} 0.150 & 0.114 & 0.119\\ 0.114 & 0.120 & 0.106\\ 0.119 & 0.106 & 0.130 \end{array}\right]$. These two variance–covariance matrices gave rise to a matrix of correlation between traits with each correlation between pairs of traits equal to 0.85. Also, we assume that the GRM is known, $G_{g}=0.7I_{80}+0.3J_{80}$, where $I_{80}$ is an identity matrix of order 80 and $J_{80}$ is a matrix of order 80×80 of ones. The relationship between environments is assumed as $\Sigma_{E}=\text{diag}(0.65,0.55,0.75)$. Therefore, the total number of observations was 3×80×3×20=14400, *i.e.*, 4800 for each trait. With these parameters, 50 data sets were simulated according to model (3) and for each data set, parameters $\beta^{T}$, $\Sigma_{t}$, $\Sigma_{E}$, and $R_{e}$ were estimated with the BMTME model using the Gibbs sampler given above. We used the priors given in the section on model implementation, which were also used for the applications with real data sets. For this simulated data set, we computed 20,000 MCMC samples, and Bayes estimates were computed with 10,000 samples, since the first 10,000 were discarded as burn-in. In Table 1, we report average estimates along with standard deviations (SD).

**Table 1:** Simulated data with three traits and three environments

	Posterior Mean of ${\hat{\Sigma }}_{t}$			Posterior SD of ${\hat{\Sigma }}_{t}$		
	T1	T2	T3	T1	T2	T3
T1	0.591	0.458	0.530	0.094	0.078	0.090
T2	—	0.500	0.488	—	0.080	0.084
T3	—	—	0.670	—	—	0.109
	Posterior Mean of ${\hat{R}}_{e}$			Posterior SD of ${\hat{R}}_{e}$		
	T1	T2	T3	T1	T2	T3
T1	0.151	0.115	0.119	0.003	0.002	0.003
T2	—	0.121	0.107	—	0.002	0.002
T3	—		0.131	—	—	0.003
	Posterior Mean of ${\hat{\Sigma }}_{E}$			Posterior SD of ${\hat{\Sigma }}_{E}$		
	E1	E2	E3	E1	E2	E3
	0.854	0.740	0.937	0.167	0.184	0.210
	Posterior Mean of $\hat{\beta }$			Posterior SD of $\hat{\beta }$		
	T1	T2	T3	T1	T2	T3
E1	15.046	8.006	7.054	0.406	0.326	0.365
E2	12.004	5.980	7.003	0.307	0.254	0.378
E3	14.104	9.003	8.053	0.464	0.407	0.434

Posterior mean and standard deviation (SD) of the β coefficients ($\hat{\beta }$) of three traits (T1, T2, and T3) in three environments (E1, E2, E3) and the estimated variance–covariance components for the traits (${\hat{\Sigma }}_{t}$), for the residuals (${\hat{R}}_{e}$), and for the environments (${\hat{\Sigma }}_{E}$).

Also, with the proposed BMTME model, we simulated two data sets similar to the simulation study explained above, except that the environmental covariance matrix we used was an identity matrix. The first data set assumes that the genetic and residual correlation between traits was 0.85 for all pairs of traits under study, while the second data set assumes that the correlation between all pairs of traits was 0.2 for both covariance matrices ($\Sigma_{t}$ and $R_{e}$). We implemented a 10-fold cross-validation (CV1). The training data set has 80% of the lines (64 lines), while the testing data set has the remaining 20% (16 lines). We assessed the prediction performance using the simulated data set under three conditions: (1) unstructured (Appendix A): assuming both variance–covariances are unstructured ($\Sigma_{t}$ and $R_{e}$); (2) diagonal (Appendix B): assuming both variance–covariances are diagonal; and (3) standard (Appendix C): assuming both variance–covariances are identity multiplied by the scale parameters $\sigma_{t}^{2}$ and $\sigma_{e}^{2}$, respectively.

### Real data sets

#### Maize data set:

A total of 309 double-haploid maize lines were phenotyped and genotyped; this is part of the data set used by Crossa *et al.* (2013) that comprised a total of 504 doubled haploid lines derived by crossing and backcrossing eight inbred lines to form several full-sib families. Traits available in this data set include grain yield (Yield), anthesis-silking interval (ASI), and plant height (PH); each of these traits was evaluated in three optimum rainfed environments (E1, E2, and E3). The experimental field design in each of the three environments was an α-lattice incomplete block design with two replicates. Data were preadjusted using estimates of block and environmental effects derived from a linear model that accounted for the incomplete block design within environment and for environmental effects.

Information about genotyping-by-sequencing (GBS) data for each maize chromosome, the number of markers after initial filtering, and the number of markers after imputation, was summarized in Crossa *et al.* (2013). Filtering was first done by removing markers that had >80% of the maize lines with missing values, and then markers with minor allele frequency lower than or equal to 0.05 were deleted. The total number of GBS data was 681,257 single nucleotide polymorphisms (SNPs) and, after filtering for missing values and minor allele frequency, 158,281 SNPs were used for the analyses. About 20% of cells were missing in the filtered GBS information used for prediction; these missing values were replaced by their expected values before doing the prediction.

#### Wheat data set:

A total of 250 wheat lines were extracted from a large set of 39 yield trials grown during the 2013–2014 crop season in Ciudad Obregon, Sonora, Mexico (Rutkoski *et al.* 2016). The trials were sown in mid-November and grown on beds with 5 and 2 irrigations plus drip irrigation. Days to heading (DTHD) were recorded as the number of days from germination until 50% of spikes had emerged in each plot, in the first replicate of each trial. Grain yield (GRYLD) was the total plot grain yield measured after maturity, and plant height (PTHT) was recorded in centimeters.

Image data of the yield trials were collected using a hyper-spectral camera (A-series, Mirco-Hyperspec VNIR, Headwall Photonics, Fitchburg, Massachusetts) mounted on a manned aircraft. From this data, vegetative indices for each plot were calculated. The green normalized difference vegetation index (GNDVI) was one of the traits used in this study. Trait GNDVI is considered a good predictor when used with pedigree and/or genomic prediction of GRYLD in wheat due to its high heritability and genetic correlation with GRYLD. Also, trait GNDVI can be measured remotely in large numbers of candidates for selection.

Genotyping-by-sequencing was used for genome-wide genotyping. Single nucleotide polymorphisms were called across all lines using the TASSEL GBS pipeline anchored to the genome assembly of Chinese Spring. Single nucleotide polymorphism calls were extracted and markers were filtered so that percent missing data did not exceed 80% and 20%, respectively. Individuals with >80% missing marker data were removed, and markers were recorded as −1, 0, and 1, indicating homozygous for the minor allele, heterozygous, and homozygous for the major allele, respectively. Next, markers with <0.01 minor allele frequency were removed, and missing data were imputed with the marker mean. A total of 12,083 markers remained after marker editing.

### Data and codes repository

The phenotypic and genotypic information of the two data sets included in this study as well as the R package for performing the analyses can be downloaded from the link: http://hdl.handle.net/11529/10646. This link contains the phenotypic data on maize (Data.maize) and wheat (Data.trigo), as well as genomic data on maize (G.maize) and wheat (G.trigo). Also, the link includes the BMTME.zip with the R package used to perform the analyses under the BMTME model.

### The BMTME R package

Nowadays the R programming language is a popular tool in statistical science for analyzing and visualizing data (R Core Team, 2015). However, in the context of big data with complex models, the speed of R is slow. For this reason, many times R is combined with C++ codes to produce high-performance programs that considerably increase the speed of programs (Stroustrup 2000; Eddelbuettel and Sanderson 2014). The R package we developed for fitting the BMTME models merges R and C++ through the use of Rcpp together with Armadillo C++ library (Sanderson 2010; Eddelbuettel 2013). Appendix E describes how the three-way data should be arranged and Appendix F explains the basic input needed to run the routines built in the R package for fitting the BMTME.

### Data availability

The authors state that all data necessary for confirming the conclusions presented in the article are represented fully within the article.

## Results

Results for the simulated data sets and for the real data sets (maize and wheat) are shown below.

### Simulated data set

Table 1 gives the posterior mean and posterior SD for β coefficients ($\beta^{T}$) for each trait and for the variance–covariance matrices ($\Sigma_{t},$ $R_{e},\Sigma_{E}$). The estimates of the posterior means for the β coefficients ($\beta^{T}$) and for the variance–covariance matrices ($\Sigma_{t},$ $R_{e}$) are very close to the true values, while the estimates of the diagonal covariance matrix ($\Sigma_{E}$) are slightly overestimated. Although the diagonal covariance matrix of $\Sigma_{E}$ is slightly overestimated according to the performed simulation study, we have evidence that the proposed BMTME model does reasonably well in terms of parameter estimation. We also tested the proposed BMTME model with another set of parameters and our results agree with the above mentioned results.

Table 2 shows the resulting prediction accuracy (Correlation and MSEP) for each environment–trait combination for the two simulated data sets; we also present the ranking of the BMTME model under the three conditions (unstructured, diagonal, and standard) for each environment–trait combination. Based on the ranking given in Table 2, the best prediction accuracy for both data sets with low and high correlation between traits (using both criteria) was achieved when the model assumed an unstructured variance–covariance matrix for both $\Sigma_{t}$ and $R_{e}$, followed by the second condition, which assumes a diagonal matrix for $\Sigma_{t}$ and $R_{e}$ in terms of MSEP, but for the standard condition in terms of the Pearson correlation. In terms of the Pearson correlation for both data sets (low and high correlation between traits), in five of the nine environment–trait combinations, the unstructured condition performed better in terms of prediction accuracy, while the standard condition performed better in three of nine environment–trait combinations, and the diagonal condition performed better in only one of nine.

**Table 2:** Simulated data with three traits and three environments

Method	E-T	Low Correlation Between Traits (0.20)						High Correlation Between Traits (0.85)					
		Correlation			MSPE			Correlation			MSPE		
		Mean	SE	R*^a^*	Mean	SE	R*^a^*	Mean	SE	R*^a^*	Mean	SE	R*^a^*
	E1-T1	0.17	0.15	2	1.27	0.15	2	0.54	0.17	1	0.96	0.21	2
	E1-T2	0.30	0.22	1	0.74	0.11	1	0.66	0.11	1	0.88	0.12	1
	E1-T3	0.51	0.12	1	0.93	0.15	1	0.59	0.09	1	1.10	0.22	1
	E2-T1	0.69	0.09	2	0.87	0.17	2	0.72	0.06	2	0.85	0.14	1
U	E2-T2	0.66	0.10	1	0.73	0.08	2	0.74	0.07	1	0.79	0.08	1
	E2-T3	0.72	0.04	1	0.79	0.14	1	0.70	0.07	2	0.99	0.18	2
	E3-T1	0.59	0.14	3	1.51	0.30	2	0.66	0.10	3	1.27	0.22	2
	E3-T2	0.80	0.06	1	0.95	0.15	1	0.77	0.07	1	1.11	0.16	1
	E3-T3	0.66	0.05	2	1.79	1.79	1	0.67	0.07	3	1.70	0.36	1
	Ave	0.57	0.11	1.56	1.06	0.34	1.44	0.67	0.09	1.67	1.07	0.19	1.33
	E1-T1	0.14	0.16	3	1.07	0.13	1	0.48	0.18	3	0.84	0.14	1
	E1-T2	0.24	0.20	3	0.78	0.07	2	0.43	0.17	3	1.01	0.11	2
	E1-T3	0.25	0.12	3	1.18	0.12	2	0.55	0.09	3	1.21	0.22	2
	E2-T1	0.71	0.07	1	0.85	0.16	1	0.76	0.04	1	0.92	0.14	2
D	E2-T2	0.64	0.07	2	0.71	0.16	1	0.70	0.05	2	0.90	0.13	2
	E2-T3	0.67	0.08	3	0.91	0.18	3	0.66	0.08	3	1.15	0.24	3
	E3-T1	0.65	0.11	2	1.26	0.35	1	0.73	0.06	2	1.19	0.27	1
	E3-T2	0.61	0.16	3	1.43	0.26	3	0.63	0.13	3	1.66	0.24	3
	E3-T3	0.66	0.04	3	2.02	2.02	3	0.69	0.07	2	1.81	0.34	3
	Ave	0.51	0.11	2.56	1.13	0.38	1.89	0.63	0.10	2.44	1.19	0.20	2.11
	E1-T1	0.22	0.17	1	1.32	0.20	3	0.53	0.18	2	1.08	0.23	3
	E1-T2	0.27	0.19	2	0.99	0.25	3	0.52	0.16	2	1.22	0.30	3
	E1-T3	0.43	0.09	2	1.23	0.18	3	0.55	0.13	2	1.48	0.38	3
	E2-T1	0.66	0.07	3	1.10	0.23	3	0.70	0.06	3	1.17	0.20	3
S	E2-T2	0.52	0.11	3	1.02	0.13	3	0.60	0.07	3	1.16	0.13	3
	E2-T3	0.71	0.07	2	0.91	0.19	2	0.73	0.07	1	0.94	0.18	1
	E3-T1	0.70	0.09	1	1.54	0.32	3	0.77	0.05	1	1.34	0.23	3
	E3-T2	0.71	0.10	2	1.03	0.18	2	0.69	0.09	2	1.17	0.18	2
	E3-T3	0.69	0.06	1	1.96	0.39	2	0.73	0.07	1	1.72	0.36	2
	Ave	0.55	0.11	1.89	1.23	0.23	2.67	0.65	0.10	1.89	1.25	0.24	2.56

Mean and standard error (SE) of the estimated correlations and Mean Squared Prediction Error (MSPE) from the 10-fold cross-validation CV1. The BMTME model was fitted using unstructured (U), diagonal (D), and standard (S) variance–covariance matrix. Environment (E1, E2, E3)–trait (T1, T2, T3) combination. Method stands for the variance–covariance matrix used with the BMTME, E-T for the environment–trait combination, R for rank, and Ave for average.

a Since three conditions are compared (unstructured, diagonal, and standard), the values of the ranks range from 1 to 3, and the lower the values, the better the prediction accuracy. For ties, we assigned the average of the ranks that would have been assigned had there been no ties.

In terms of MSEP, the unstructured BMTME performed better in five (low correlation between traits) and six (high correlation between traits) of the nine environment–trait combinations, the diagonal BMTME in only four (low correlation between traits) and two (high correlation between traits) of nine combinations, and the standard BMTME in zero (low correlation between traits) and one (high correlation between traits) of nine combinations. Regarding the average of the nine groups (environment–trait combinations) for both prediction criteria (correlation and MSEP), the unstructured BMTME gave the best prediction, correlation = 0.57 (low correlation between traits), and correlation = 0.67 (high correlation between traits) and MSEP = 1.06 (low correlation between traits) and MSEP = 1.07 (high correlation between traits). In both data sets, the unstructured BMTME model had the best prediction accuracy; however, the higher the correlation between traits, the higher the prediction accuracies observed, since the average correlation between traits under the unstructured BMTME was 17.5% higher when the correlation between traits was 0.85 compared to when it was 0.2.

### Maize data set

Table 3 shows that for each trait there are moderate differences between the β coefficients between environments. For Yield and PH, the largest and smallest β coefficients were observed in environments E1 and E2, respectively, while for trait ASI, the largest β coefficient was observed in E3 and the smallest in E2. The genetic estimates of the variance–covariance components of traits are given in ${\hat{\Sigma }}_{t}$, where the correlation between traits is moderate. Yield and ASI have a negative correlation (−0.27), and the correlation between ASI and PH is also negative (−0.25), while the correlation between Yield and PH is 0.41. The same tendency is observed in the residual correlation between traits but with smaller correlation between traits.

**Table 3:** Maize data

	Posterior Mean of ${\hat{\Sigma }}_{t}$			Posterior SD of ${\hat{\Sigma }}_{t}$		
	Yield	ASI	PH	Yield	ASI	PH
Yield	1.666	−0.260	0.069	0.430	0.210	0.030
ASI	−0.158	1.631	−0.046	—	0.430	0.030
PH	0.315	−0.212	0.028	—	—	0.010
	Posterior Mean of ${\hat{R}}_{e}$			Posterior SD of ${\hat{R}}_{e}$		
	Yield	ASI	PH	Yield	ASI	PH
Yield	0.506	−0.077	0.022	0.050	0.030	0.000
ASI	−0.151	0.512	−0.012	—	0.050	0.000
PH	0.278	−0.153	0.013	—	—	0.000
	Posterior Mean of ${\hat{\Sigma }}_{E}$			Posterior SD of ${\hat{\Sigma }}_{E}$		
	E1	E2	E3	E1	E2	E3
	0.663	0.655	0.898	0.0369	0.0320	0.0349
	Posterior Mean of $\hat{\beta }$			Posterior SD of $\hat{\beta }$		
	Yield	ASI	PH	Yield	ASI	PH
E1	6.445	1.872	2.354	0.210	0.280	0.030
E2	4.958	1.147	2.066	0.280	0.330	0.040
E3	6.102	2.276	2.341	0.290	0.300	0.040
	Correlation in the Entire Data			MSPE in the Entire Data		
	Yield	ASI	PH	Yield	ASI	PH
E1	0.796	0.756	0.646	0.428	0.233	0.012
E2	0.769	0.798	0.757	0.245	0.605	0.007
E3	0.799	0.794	0.763	0.480	0.338	0.011

Posterior mean and SD of the β coefficients ($\hat{\beta }$) for three traits, Yield, anthesis-silking interval (ASI), and plant height (PH) in three environments (E1, E2, and E3). Estimate variance–covariance components for the traits (${\hat{\Sigma }}_{t}$), the environments (${\hat{\Sigma }}_{E}$), and the residuals (${\hat{R}}_{e}$). In ${\hat{\Sigma }}_{t}$ and ${\hat{R}}_{e}$, the upper triangle contains the variance–covariance components and the lower triangle contains the correlations. ${\hat{\Sigma }}_{E}$ is a diagonal matrix.

Table 4 shows the prediction accuracies (Correlation and MSEP) for each environment–trait combination and the ranking of the three conditions studied for each criterion in the maize testing data set for cross-validation CV1. From the ranking, the best condition is the standard model, since it was the best in five of nine environment–trait combinations in terms of correlation, while in terms of MSEP, the diagonal model was the best in three of nine environment–trait combinations. As for the averages of the environment–trait combinations, the standard model was also the best in terms of both criteria. The second-best model was the diagonal, and the unstructured model was the worst in terms of both criteria. This can be explained by the low correlation between traits that exists for this maize data set.

**Table 4:** Maize data

		Correlation			MSPE		
BMTME	Environment–Trait	Mean	SE	Rank*^a^*	Mean	SE	Rank
	E1-Yield	0.28	0.07	3	0.74	0.08	3.00
	E2-Yield	0.40	0.09	1.5	0.39	0.06	2.50
	E3-Yield	0.37	0.08	2.5	0.02	0.01	1.50
	E1-ASI	0.39	0.08	2	0.37	0.03	1.50
Unstructured	E2-ASI	0.46	0.06	2.5	1.26	0.35	3.00
	E3-ASI	0.42	0.05	2	0.01	0.00	1.50
	E1-PH	0.37	0.06	2	0.86	0.07	3.00
	E2-PH	0.26	0.08	3	0.48	0.07	3.00
	E3-PH	0.44	0.07	2.5	0.02	0.00	2.00
	Average	0.37	0.07	2.33	0.46	0.08	2.33
	E1-Yield	0.30	0.07	1.5	0.73	0.07	2.00
	E2-Yield	0.40	0.08	1.5	0.36	0.03	1.00
	E3-Yield	0.37	0.05	2.5	0.85	0.07	3.00
	E1-ASI	0.40	0.09	1	0.39	0.06	3.00
Diagonal	E2-ASI	0.46	0.06	2.5	1.25	0.35	2.00
	E3-ASI	0.27	0.08	3	0.48	0.07	3.00
	E1-PH	0.36	0.07	3	0.02	0.01	1.00
	E2-PH	0.41	0.06	1	0.01	0.00	1.00
	E3-PH	0.44	0.06	2.5	0.02	0.00	2.00
	Average	0.38	0.07	2.06	0.46	0.08	2.00
	E1-Yield	0.30	0.07	1.5	0.72	0.07	1.00
	E2-Yield	0.39	0.09	3	0.39	0.06	2.50
	E3-Yield	0.38	0.08	1	0.02	0.01	1.50
	E1-ASI	0.38	0.08	3	0.37	0.03	1.50
Standard	E2-ASI	0.48	0.06	1	1.24	0.35	1.00
	E3-ASI	0.43	0.05	1	0.01	0.00	1.50
	E1-PH	0.39	0.06	1	0.84	0.06	2.00
	E2-PH	0.27	0.08	2	0.47	0.07	2.00
	E3-PH	0.45	0.07	1	0.02	0.00	2.00
	Average	0.39	0.07	1.61	0.45	0.07	1.67

Mean and SE of the estimated correlations and MSPE from the 10-fold cross-validation CV1. The BMTME model was fitted using unstructured, diagonal, and standard variance–covariance matrices. Environment (E1, E2, E3)–trait (Yield, ASI, PH) combination.

a Since three BMTME models are fitted (unstructured, diagonal, and standard) the values of the ranks ranged from 1 to 3, and the lower the values, the better the prediction accuracy. For ties, we assigned the average of the ranks that would have been assigned had there been no ties.

Table 5 provides the results of cross-validation CV2 for the maize testing data set. The trait yield is unobserved in only one environment (for example, E1) for all lines, but data on the other two traits are available for this environment (E1), as well as for the other two environments (E2 and E3). The best model in terms of correlation and MSEP for predicting Yield for all lines in E1 was the standard model (0.215, 41.714), followed by the diagonal (0.168, 43.691) and the unstructured model (0.163, 46.407). In E2-Yield and E3-Yield, the best BMTME model was the unstructured model with Pearson correlation. In terms of MSEP, the BMTME unstructured model was the best model for predicting the Yield of the unobserved lines in E2, followed by the other two models (diagonal and standard). For E3-Yield, the best predictive model in terms of MSEP was the BMTME standard, followed by the unstructured model.

**Table 5:** Maize data

	Unstructured		Diagonal		Standard	
Environment–Trait	Correlation	MSPE	Correlation	MSPE	Correlation	MSPE
E1-Yield	0.163	46.407	0.168	43.691	0.215	41.714
E2-Yield	0.405	23.671	0.156	31.586	0.214	24.943
E3-Yield	0.298	39.946	0.243	42.735	0.247	37.383

Mean of the estimated correlations and MSPE for predicting the trait Yield for all lines in each environment. The BMTME was fitted using unstructured, diagonal, and standard variance–covariance matrices. Environment (E1, E2, and E3)–trait Yield.

### Wheat data set

Table 6 shows that β coefficients are very different between traits and environments for the wheat data set. In environment Bed2IR, the largest β coefficients were observed in traits DTHD and GNDVI, respectively, while in environment Drip, the largest β coefficients were observed in traits GNDVI and GRYLD, respectively. The genetic estimates of the variance–covariance components of traits are given in ${\hat{\Sigma }}_{t}$,where the largest correlations were observed between trait DTHD *vs.* GNDVI, GRYLD, and PTHT; the same is true for the residual correlation between traits (${\hat{R}}_{e}$). In terms of prediction accuracies for the entire data set, they are high in terms of correlation and less precise in terms of MSEP mostly for trait PTHT in the three environments.

**Table 6:** Wheat data

	Posterior Mean of ${\hat{\Sigma }}_{t}$				Posterior SD of ${\hat{\Sigma }}_{t}$			
	DTHD	GNDVI	GRYLD	PTHT	DTHD	GNDVI	GRYLD	PTHT
DTHD	16.172	0.028	−0.413	−4.505	0.696	0.002	0.047	0.619
GNDVI	0.7348	0.000	0.000	−0.010	—	0.000	0.000	0.002
GRYLD	−0.386	−0.19	0.071	0.111	—	—	0.008	0.061
PTHT	−0.386	−0.35	0.144	8.442	—	—	—	1.023
	Posterior Mean of ${\hat{R}}_{e}$				Posterior SD of ${\hat{R}}_{e}$			
	DTHD	GNDVI	GRYLD	PTHT	DTHD	GNDVI	GRYLD	PTHT
DTHD	0.523	−0.003	0.112	0.606	0.214	0.001	0.035	0.393
GNDVI	−0.453	0.000	0.000	−0.002	—	0.000	0.000	0.002
GRYLD	0.569	−0.192	0.074	0.561	—	—	0.008	0.077
PTHT	0.215	−0.048	0.530	15.214	—	—	—	1.327
	Posterior Mean of ${\hat{\Sigma }}_{E}$				Posterior SD of ${\hat{\Sigma }}_{E}$			
	Bed2IR	Bed5IR	Bed5IR		Bed2IR	Bed5IR	Bed5IR	
	0.461	1.326	0.014	—	0.076	0.189	0.020	—
	Posterior Mean of $\hat{\beta }$				Posterior SD of $\hat{\beta }$			
	DTHD	GNDVI	GRYLD	PTHT	DTHD	GNDVI	GRYLD	PTHT
Bed2IR	−3.202	−4.061	−0.312	−0.004	0.227	0.233	0.223	0.001
Bed5IR	−0.011	0.006	−0.135	−0.341	0.001	0.001	0.023	0.024
Drip	−0.407	−4.595	−7.368	−0.576	0.023	0.292	0.295	0.291
	Correlation in the Entire Data				MSPE in the Entire Data			
	DTHD	GNDVI	GRYLD	PTHT	DTHD	GNDVI	GRYLD	PTHT
Bed2IR	0.999	0.930	0.906	0.906	0.115	0.000	0.018	10.187
Bed5IR	0.998	0.943	0.885	0.666	0.227	0.000	0.059	8.284
Drip	0.992	0.908	0.873	0.871	0.352	0.000	0.069	13.839

Posterior mean and SD of the β coefficients ($\hat{\beta }$) for four traits (DTHD, GNDVI, GRYLD, and PTHT) in three environments (Bed2I, Bed5I, and Drip). Estimated variance–covariance components for traits (${\hat{\Sigma }}_{t}$) and for residual (${\hat{R}}_{e}$). In ${\hat{\Sigma }}_{t}$, and ${\hat{R}}_{e}$ the upper triangle contains the variance–covariance components and the lower triangle contains the correlations.

Table 7 gives the prediction accuracy of the wheat data set for the testing data set for each environment–trait combination; it also gives the ranking of the three conditions studied under both criteria for cross-validation CV1. The best case is when the BMTME model assumes an unstructured variance–covariance matrix for both $\Sigma_{t}$ and $R_{e}$ and a diagonal matrix for the variance–covariance for $\Sigma_{E},$ followed by BMTME with a diagonal matrix for $\Sigma_{t,}$ $R_{e}$, and $\Sigma_{E}.$ As for the ranking in terms of Pearson correlation, in 6 of 12 groups the BMTME unstructured model performed better in terms of prediction accuracy, while the BMTME diagonal model was the second-best model since it was the best model in 3 of 12 cases; the BMTME standard model was the worst model in terms of prediction accuracy since it was the best in only 1 of 12 cases. In terms of MSEP, the BMTME unstructured model performed better in 5 of 12 cases, the BMTME diagonal model was the best model in only 3 of 12 cases, and the BMTME standard model was the best in 1 of 12 cases. The BMTME unstructured model also had the best average prediction accuracy of the 12 groups.

**Table 7:** Wheat data

Method	Environment–trait	Correlation			MSEP		
		Mean	SE	Rank*^a^*	Mean	SE	Rank
	Bed2I-DTHD	0.93	0.03	2	4.82	1.60	2
	Bed2I-GNVI	0.79	0.08	1.5	6.8E-05	0.00	2
	Bed2I-GRYLD	0.64	0.12	1	0.05	0.01	1
	Bed2I-PTHT	0.60	0.18	1	26.55	11.90	2
	Bed5I-DTHD	0.76	0.13	2	17.61	7.63	1
U	Bed5I-GNVI	0.60	0.20	1	9.7E-05	0.00	2
	Bed5I-GRYLD	0.35	0.32	2	0.23	0.09	2
	Bed5I-PTHT	0.46	0.16	3	11.90	3.40	3
	Drip-DTHD	0.95	0.02	1	2.66	0.81	1
	Drip-GNVI	0.68	0.21	1.5	0.00	0.00	2
	Drip-GRYLD	0.67	0.17	1	0.13	0.05	1
	Drip-PTHT	0.69	0.08	1	22.68	10.21	1
	Ave	0.68	0.14	1.50	7.22	2.98	1.67
	Bed2I-DTHD	0.95	0.01	1	4.44	0.57	1
	Bed2I-GNVI	0.79	0.01	1.5	6.3E-05	0.00	2
	Bed2I-GRYLD	0.60	0.04	2	0.06	0.00	2
	Bed2I-PTHT	0.56	0.06	2	28.24	3.70	2
	Bed5I-DTHD	0.79	0.04	1	16.51	2.37	1
D	Bed5I-GNVI	0.66	0.06	2	8.4E-05	0.00	2
	Bed5I-GRYLD	0.38	0.09	1	0.22	0.02	1
	Bed5I-PTHT	0.47	0.06	2	11.86	1.10	2
	Drip-DTHD	0.94	0.01	2	4.34	0.53	2
	Drip-GNVI	0.68	0.06	1.5	0.00	0.00	2
	Drip-GRYLD	0.59	0.06	3	0.14	0.02	2
	Drip-PTHT	0.62	0.03	2	23.83	3.14	2
	Ave	0.67	0.04	1.75	7.47	0.95	1.75
	Bed2I-DTHD	0.94	0.05	3	17.37	6.94	3
	Bed2I-GNVI	0.33	0.21	3	0.00	0.00	2
	Bed2I-GRYLD	0.58	0.18	3	0.07	0.01	3
	Bed2I-PTHT	0.56	0.23	3	32.70	14.42	3
	Bed5I-DTHD	0.78	0.14	3	30.94	8.83	3
S	Bed5I-GNVI	0.46	0.25	3	0.00	0.00	2
	Bed5I-GRYLD	0.38	0.33	3	0.24	0.09	3
	Bed5I-PTHT	0.41	0.18	1	9.88	3.18	1
	Drip-DTHD	0.93	0.05	3	7.27	2.56	3
	Drip-GNVI	0.43	0.14	3	0.00	0.00	2
	Drip-GRYLD	0.55	0.18	2	0.17	0.08	3
	Drip-PTHT	0.61	0.16	3	28.84	12.68	3
	Ave	0.58	0.17	2.75	10.62	4.07	2.58

Mean and SE of the estimated correlations and MSPE from the 10-fold cross-validation CV1. The BMTME model was fitted using unstructured (U), diagonal (D), and standard (S) variance–covariance matrices. Environment (Bed2I, Bed5I, Drip)–trait [days to heading (DTHD), GNDVI, grain yield (GRYLD), and plant height (PTHT)] combination. Method stands for the three variance–covariance matrices used with the BMTME.

a Since three BMTME models are fitted (unstructured, diagonal, and standard), the values of the ranks ranged from 1 to 3, and the lower the values, the better the prediction accuracy. For ties, we assigned the average of the ranks that would have been assigned had there been no ties.

Table 8 gives the results of cross-validation CV2 which assumes that trait GRYLD is lacking in one environment for all lines but not in the other environments. The results given are only for the testing data set (trait GRYLD missing for all lines in one environment). The best model for predicting GRYLD for all lines in environment Bed2I with Pearson correlation was the BMTME unstructured model, followed by the BMTME standard and, in the last position, the BMTME diagonal model. In terms of MSEP, the results are exactly the opposite. While in environment Bed5I the best predictive model in terms of Pearson correlation was the BMTME standard, then the BMTME diagonal, and, in the last position, the BMTME unstructured model. In terms of MSEP, the best model was the unstructured model, then the standard, and, at the end, the diagonal model. In environment Drip, the ranking of models based on both criteria was as follows: BMTME unstructured, BMTME diagonal, and BMTME standard.

**Table 8:** Wheat data

	Unstructured		Diagonal		Standard	
Environment–trait	Correlation	MSEP	Correlation	MSEP	Correlation	MSEP
Bed2I-GRYLD	0.648	0.085	0.589	0.079	0.580	0.076
Bed5I-GRYLD	0.173	0.342	0.164	0.408	0.187	0.343
Drip-GRYLD	0.634	0.246	0.516	0.264	0.420	0.304

Mean of the estimated correlations and MSPE for the prediction of the trait grain yield (GRYLD) for all lines in each environment (Bed2I, Bed5I, Drip). The BMTME was fitted using unstructured, diagonal, and standard variance–covariance matrices.

## Discussion

To our knowledge, this is the first statistical three-way genomic model for assessing the prediction accuracy of trait × genotype × environment. Other models for assessing multi-traits or multi-environments have been extensively studied in the related literature (see, for example, Jarquín *et al.* 2014; Montesinos-López *et al.* 2015); however, none of them have simultaneously assessed and modeled the three-way variance–covariance structure. The BMTME model does this task simultaneously using Bayesian estimation and the package for performing such a task is given in this article.

### Performance of the BMTME model in simulated and real data sets

In the simulated data sets, the best prediction accuracies were achieved with the BMTME model (which assumes an unstructured variance–covariance matrix for the genetic and residual components) even when the correlation between traits was low, followed by the model that assumed a diagonal variance–covariance matrix for both matrices (in terms of the Pearson correlation) of traits, and then by the standard model, which was formed by an identity matrix multiplied by $\sigma_{t}^{2}$ and $\sigma_{e}^{2}$ for the genetic and residual variance–covariance matrices, respectively. The simulation study provides evidence that when the correlation between traits is high, it is really important to use a multivariate model that takes into account this correlation to improve prediction accuracies.

This evidence is also supported by the results obtained with the wheat data set, where the BMTME unstructured model was the most accurate model, followed by the diagonal and finally by the standard model. However, with the maize data set, we did not observe any gain using the unstructured variance–covariance matrix in comparison to the other two variance–covariances used (diagonal and standard), maybe because in this data set the genetic and residual correlations between traits were low. Therefore, the important message is that when the correlation between traits is high (>0.5), it is really important to estimate the unstructured variance–covariance matrix; when this correlation is low, it is enough to use the BMTME standard model because with the unstructured model, the results could be worse than those of the standard model. These suggestions are not new; they were also made by Calus and Veerkamp (2011), Jia and Jannink (2012), Guo *et al.* (2014), and Jiang *et al.* (2015) in the context of multi-trait analysis. Here we only point out that they are also valid in the multi-trait, multi-environment context, taking into account the T × G × E interaction term.

Our contribution added to the traditional multi-trait model (proposed by Calus and Veerkamp 2011; Jia and Jannink 2012; Guo *et al.* 2014; Jiang *et al.* 2015) is that our model also is valid for the multi-environment and the three-way (T × G × E) interaction term, which more realistically mimic the type of data that are very common in plant breeding programs, where genotypes are evaluated for multi-traits in multi-environments. We are also aware that normally distributed traits are not the only traits commonly measured in plant breeding programs. For this reason, models for multiple categorical ordinal traits, multiple count traits, or a mixture of types of traits are also needed to help breeders improve the process of selecting candidate genotypes.

### Prediction assessment of the BMTME model

We introduced a Gibbs sampler for Bayesian analysis of multi-traits and multi-environments that takes into account the three-way (T × G × E) interaction term that uses simple conditional distribution to simulate the joint posterior distribution of all required unknown parameters in the WGP model. This model has the advantage that it uses Half-t priors on each standard deviation term and uniform priors between −1 and 1 on each correlation of the covariance matrix of traits in order to achieve noninformativity and posterior inferences with low sensitivity to the choice of hyper-parameters for the variance–covariance matrices.

Since we modeled the correlation patterns separately for each factor as $G_{1}=G_{g}⊗\Sigma_{t}$ and $G_{2}=\Sigma_{E}⊗G_{g}⊗\Sigma_{t}$, this facilitates the interpretation of the contribution of every factor to the overall correlation structure. It also allows choosing specific covariance structures for each factor, which improves accuracy and makes model fitting easier. In addition, fewer parameters than an unstructured model are required. For example, for modeling $G_{2}$ under an unstructured model, we need to estimate IJL(IJL+1)/2 unknown parameters; this number of parameters is larger than the number of parameters required to be estimated using Kronecker products for a three-factor separable model that only needs $\frac{I\left(I+1\right)}{2}+\frac{J\left(J+1\right)}{2}+\frac{L\left(L+1\right)}{2}-2$ parameters. In our context, the number of parameters is lower, since we assumed a diagonal matrix for the variance–covariance matrix of environments and the matrix $G_{g}$ is given. Also, if needed, partial derivatives, inverse computation, and Cholesky decomposition of the overall covariance matrix are performed more easily on the factor-specific covariances because they have smaller dimensions. Therefore, the use of separable covariance matrices with Kronecker products has substantial computational advantages, besides improving interpretation and model fitting (Simpson *et al.* 2014). However, care needs to be exercised with the assumption of a Kronecker product structured variance–covariance matrix, especially in three-way multivariate data, because incorrect assumptions may lead to invalid conclusions (Roy and Leiva 2008).

### Contributions and limitations of the BMTME model

This study clearly described the full conditional distributions for modeling the three-way (T × G × E) interaction term with multi-traits and multi-environments, which is of paramount importance for evaluating genotypic performance in target environments and for predicting yet-to-be observed phenotypes when the relative performance of genotypes varies across environments. Because the proposed model takes into account the correlation between traits and includes the three-way (T × G × E) interaction term, the BMTME can be a useful tool for efficiently selecting superior genotypes. The proposed BMTME model can be considered a Bayesian GBLUP for multiple traits and multiple environments since the marker information is taken into account in the GRM ($G_{g}$). Some of the advantages of our model over standard software are: (a) it is able to estimate separable covariance matrices of the form A⊗B⊗C, which is not possible with other software; (b) the estimation of three-way terms with covariance matrices of the form A⊗B⊗C is more parsimonious since fewer parameters are needed than when two factors are joined and the estimation process is performed using two separable covariance structures as $A⊗B^{*}$, where $B^{*}$ contains the covariance of the two factors ***B*** and C; (c) the convergence of our model is not a big deal compared to the convergence problems of other software for complex data; and (d) our model facilitates the interpretation of the covariance matrices because we can estimate the three covariance matrices.

On the other hand, as expected, the disadvantage of the BMTME model is its high computational cost even under the optimized C++ developed and made available in this research article. Large numbers of lines might indeed cause some delays in the computation of such large numbers of parameters in the full conditionals. However, constant developments in computing science will soon reduce the computing time of the three-way BMTME model.

Finally, our proposed BMTME model can also be useful (a) in QTL-mapping studies, since some WGP methods are also commonly used for GWAS (Peters *et al.* 2012; Garrick and Fernando, 2013; Jiang *et al.* 2015), and (b) to include spatial information in the residual R matrix of the proposed model. This information is often available from breeding programs since they measure geographical information of the plots where genotypes are tested in each environment; this could help improve prediction accuracy.

### Conclusions

In this paper, we extended the multi-trait WGP model to the multi-trait and multi-environment WGP model. This unified WGP model takes into account the correlation between traits and the three-way interaction term (T × G × E). Additionally, a transparent derivation of all full conditional distributions required is given that allows us to propose an efficient Gibbs sampler that is easy to implement and produces precise parameter estimates with high noninformativity and posterior inferences with low sensitivity to the choice of hyper-parameters for the variance–covariance matrices. Finally, we successfully applied the proposed method to simulated and real data and found that when the correlation between the traits is high (>0.5), the proposed BMTME model with an unstructured covariance matrix should be preferred over the diagonal and standard methods to help improve prediction accuracy. However, when correlations are low, it is enough to use the BMTME standard model because if we use the unstructured model, the results could be worse than those of the standard model. The R-software package BMTME offers specialized and optimized C++ routines to efficiently perform the analyses under the proposed model.

## Appendix A

### Derivation of full conditional distributions for the BMTME unstructured model

#### Full conditional for β

$$\begin{array}{c} P\left(\beta |ELSE\right)=P\left(Y|\beta ,b_{1},b_{2},R_{e}\right)P\left(\beta |\sigma_{\beta }^{2}\right)\\ \propto \exp\left(-\frac{1}{2}{\left(Y-\mathrm{X\beta}-\sum_{h=1}^{2}Z_{h}b_{h}\right)}^{T}R^{-1}\left(Y-\mathrm{X\beta}-\sum_{h=1}^{2}Z_{h}b_{h}\right)-\frac{1}{2}{\left(\beta -\beta_{0}\right)}^{T}\Sigma_{0}^{-1}\sigma_{\beta }^{-2}\left(\beta -\beta_{0}\right)\right)\\ \propto \exp\left(-\frac{1}{2}\left[{\left(\beta -{\tilde{\beta }}_{0}\right)}^{T}{\tilde{\Sigma }}_{0}^{-1}\left(\beta -{\tilde{\beta }}_{0}\right)\right]\right)\propto N\left({\hat{\beta }}_{0},{\tilde{\Sigma }}_{0}\right) \end{array}$$ (A.1)

where ${\tilde{\Sigma }}_{0}={\left(\Sigma_{0}^{-1}\sigma_{\beta }^{-2}+X^{T}R^{-1}X\right)}^{-1}$, ${\tilde{\beta }}_{0}={\tilde{\Sigma }}_{0}\left(\Sigma_{0}^{-1}\sigma_{\beta }^{-2}\beta_{0}-X^{T}R^{-1}\overset{2}{\underset{h=1}{\Sigma }}Z_{h}b_{h}+X^{T}R^{-1}Y\right)$ with $R^{-1}=I_{n}⊗R_{e}^{-1}$. Also, if we had assumed P(β)∝1 as prior for β, we would have maintained a multivariate Normal posterior distribution due to the multivariate Normal distribution’s conjugacy. However, the mean vector and covariance matrix would be slightly modified.

#### Full conditional for $\sigma_{\beta }^{2}$

$$\begin{array}{l} P\left(\sigma_{\beta }^{2}|\mathrm{ELSE}\right)\propto P\left(\beta |\sigma_{\beta }^{2}\right)P\left(\sigma_{\beta }^{2}|a_{\beta }\right)\\ \propto \frac{1}{{\left(\sigma_{\beta }^{2}\right)}^{\frac{\nu_{\beta }+1+IL+1}{2}}}\exp\left(-\frac{{\left(\beta -\beta_{0}\right)}^{T}\Sigma_{0}^{-1}\left(\beta -\beta_{0}\right)+2\nu_{\beta }/a_{\beta }}{2\sigma_{\beta }^{2}}\right)\\ \propto IW\left({\tilde{k}}_{\beta^{*}}=\nu_{\beta }+IL,{\tilde{B}}_{\beta }={\left(\beta -\beta_{0}\right)}^{T}\Sigma_{0}^{-1}\left(\beta -\beta_{0}\right)+2\nu_{\beta }/a_{\beta }\right) \end{array}$$ (A.2)

#### Full conditional for $a_{\beta }$

$$\begin{array}{l} P\left(a_{\beta }|\mathrm{ELSE}\right)\propto P\left(\sigma_{\beta }^{2}|a_{\beta }\right)P\left(a_{\beta }\right)\\ \propto \frac{1}{{\left(a_{\beta }\right)}^{\frac{\nu_{\beta }+1}{2}+1}}\exp\left(-\frac{1/A_{\beta }^{2}+\nu_{\beta }/\sigma_{\beta }^{2}}{a_{\beta }}\right)\\ \propto IG\left(\frac{\nu_{\beta }+1}{2},1/A_{\beta }^{2}+\nu_{\beta }/\sigma_{\beta }^{2}\right) \end{array}$$ (A.3)

#### Full conditional for $b_{1}$

Defining $\eta^{1}=\mathrm{X\beta}+Z_{2}b_{2},$ the conditional distribution of $b_{1}$ is given as

$$P\left(b_{1}\text{|}ELSE\right)\propto \;P(b_{1}|\Sigma_{t})P(Y|\beta ,b_{1},b_{2},R_{e})$$

$$\propto \exp\left(-\frac{1}{2}{\left(Y-Z_{1}b_{1}-\eta^{1}\right)}^{T}R^{-1}\left(Y-Z_{1}b_{1}-\eta^{1}\right)-\frac{1}{2}b_{1}^{T}G_{1}^{-1}b_{1}\right)$$

$$\propto \exp\left\{-\frac{1}{2}{\left(b_{1}-{\tilde{b}}_{1}\right)}^{T}F_{1}^{-1}(b_{1}-{\tilde{b}}_{1})\right\}\propto N\left({\tilde{b}}_{1},F_{1}\right)$$ (A.4)

where $F_{1}={\left(G_{1}^{-1}+Z_{1}^{T}R^{-1}Z_{1}\right)}^{-1}\text{ and }{\tilde{b}}_{1}=F_{1}\left(Z_{1}^{T}R^{-1}Y-Z_{1}^{T}R^{-1}\eta^{1}\right)$. In a similar way, by defining $\eta^{2}=\mathrm{X\beta}+Z_{1}b_{1}$, we arrive at the full conditional of $b_{2}$ as

$$P\left(b_{2}|\mathrm{ELSE}\right)∼N\left({\tilde{b}}_{2},F_{2}\right)$$ (A.5)

where $F_{2}={\left(G_{2}^{-1}+Z_{2}^{T}R^{-1}Z_{2}\right)}^{-1},{\tilde{b}}_{2}=F_{2}\left(Z_{2}^{T}R^{-1}Y-Z_{2}^{T}R^{-1}\eta^{2}\right),$$G_{1}^{-1}=G_{g}^{-1}⊗\Sigma_{t}^{-1}$, and $G_{2}^{-1}=\Sigma_{E}^{-1}⊗G_{1}^{-1}$.

#### Full conditional for $\Sigma_{t}$

$$P\left(\Sigma_{t}|ELSE\right)\propto P(b_{1}|\Sigma_{t})P(\Sigma_{t}|a_{1,}\ldots ,a_{L})P(b_{2}|\Sigma_{t},\Sigma_{E})\propto {\left|G_{g}⊗\Sigma_{t}\right|}^{-\frac{1}{2}}\exp\left(-\frac{1}{2}b_{1}^{T}{\left(G_{g}⊗\Sigma_{t}\right)}^{-1}b_{1}\right)P(\Sigma_{t}|a_{1,}\ldots ,a_{L}){\left|G_{3}⊗\Sigma_{t}\right|}^{-\frac{1}{2}}\exp\left(-\frac{1}{2}b_{2}^{T}{\left(G_{3}⊗\Sigma_{t}\right)}^{-1}b_{2}\right)$$

with $G_{3}=\Sigma_{E}⊗G_{g}$

$$\propto {\left|\Sigma_{t}\right|}^{-\frac{J+JI}{2}}\exp\left(-\frac{1}{2}[b_{1}^{T}\left(G_{g}^{-\frac{1}{2}}⊗\Sigma_{t}^{-\frac{1}{2}}\right)\left(G_{g}^{-\frac{1}{2}}⊗\Sigma_{t}^{-\frac{1}{2}}\right)b_{1}+b_{2}^{T}\left(G_{3}^{-\frac{1}{2}}⊗\Sigma_{t}^{-\frac{1}{2}}\right)\left(G_{3}^{-\frac{1}{2}}⊗\Sigma_{t}^{-\frac{1}{2}}\right)b_{2}]\right)P(\Sigma_{t}|a_{1,}\ldots ,a_{L})$$

$$\propto {\left|\Sigma_{t}\right|}^{-\frac{J+JI}{2}}\exp\left(-\frac{1}{2}[\overset{J}{\underset{j=1}{\sum }}c_{1j}^{T}\Sigma_{t}^{-\frac{1}{2}}\Sigma_{t}^{-\frac{1}{2}}c_{1j}+\overset{JL}{\underset{j*=1}{\sum }}c_{2j*}^{T}\Sigma_{t}^{-1/2}\Sigma_{t}^{-1/2}c_{2j*}\right)P(\Sigma_{t}|a_{1,}\ldots ,a_{L})$$

$$\propto {\left|\Sigma_{t}\right|}^{-\frac{\nu_{t}+J+L+JI-1+L+1}{2}}\exp\left(-\frac{1}{2}(tr\{[b_{1}^{*}G_{g}^{-1}b_{1}^{*T}+b_{2}^{*}G_{3}^{-1}b_{2}^{*T}+B_{t}]\Sigma_{t}^{-1}\})\right){\left|B_{t}\right|}^{\frac{\nu_{t}+L-1}{2}}$$

$\propto IW(\kappa^{*}=\nu_{t}+J+L+JI-1,B_{t}^{*}=[b_{1}^{*}G_{g}^{-1}b_{1}^{*T}+b_{2}^{*}G_{3}^{-1}b_{2}^{*T}+2\nu_{t}\text{diag}(\frac{1}{a_{1}},\ldots ,\frac{1}{a_{L}})$]) (A.6) where $B_{t}=2\nu_{t}\text{diag}(\frac{1}{a_{1}},\ldots ,\frac{1}{a_{L}})$. Note that $(G_{g}^{-1/2}⊗\Sigma_{t}^{-1/2})b_{1}=vec\left(\Sigma_{t}^{-\frac{1}{2}}b_{1}^{*}G_{g}^{-\frac{1}{2}}\right)=vec\left(\Sigma_{t}^{-\frac{1}{2}}C_{1}\right)$, with $b_{1}^{*}=[b_{11},\ldots ,b_{1J}]$, $C_{1}=[c_{11},\ldots ,c_{1J}]=b_{1}^{*}G_{g}^{-1/2}$. From here, $(G_{g}^{-1/2}⊗\Sigma_{t}^{-1/2})b_{1}=vec\left(\left[\Sigma_{t}^{-\frac{1}{2}}c_{11},\ldots ,\Sigma_{t}^{-\frac{1}{2}}c_{1J}\right]\right)=\left[\begin{array}{c} \Sigma_{t}^{-\frac{1}{2}}c_{11}\\ \vdots \\ \Sigma_{t}^{-\frac{1}{2}}c_{1J} \end{array}\right]$, and so $b_{1}^{T}{\left(G_{g}⊗\Sigma_{t}\right)}^{-1}b_{1}=\overset{J}{\underset{j=1}{\Sigma }}c_{1j}{}^{T}\Sigma_{t}^{-1}c_{1j}=tr\left[\left(\overset{J}{\underset{j=1}{\Sigma }}C_{1}u_{j}u_{j}{}^{T}C_{1}{}^{T}\right)\Sigma_{t}^{-1}\right]=tr\left[\left(C_{1}\overset{J}{\underset{j=1}{\sum }}u_{j}u_{j}{}^{T}C_{1}{}^{T}\right)\Sigma_{t}^{-1}\right]=tr\left[\left(C_{1}C_{1}{}^{T}\right)\Sigma_{t}^{-1}\right]=tr\left[b_{1}^{*}G_{g}^{-1}b_{1}^{*T}\Sigma_{t}^{-1}\right].$ Also note that $(G_{3}^{-1/2}⊗\Sigma_{t}^{-1/2})b_{2}=vec\left(\Sigma_{t}^{-\frac{1}{2}}b_{2}^{*}G_{3}^{-\frac{1}{2}}\right)=vec\left(\Sigma_{t}^{-\frac{1}{2}}C_{2}\right)$ with $b_{2}^{*}=[b_{21},\ldots ,b_{2JI}]$, $C_{2}=\left[c_{21},\ldots ,c_{2JL}\right]=b_{2}^{*}G_{3}^{-1/2}$. Obtained using $\left(B^{T}⊗A)vec(X)=vec(\mathrm{AXB}\right)$.

#### Full conditional for $a_{l}$

$$\begin{array}{l} P\left(a_{l}|\mathrm{ELSE}\right)\propto P\left(\Sigma_{t}|a_{l}\right)P\left(a_{l}\right)\\ \propto \frac{1}{{\left(a_{l}\right)}^{\frac{\nu_{t}+L}{2}+1}}\exp\left(-\frac{1/A_{l}^{2}+\nu_{t}{\left(\Sigma_{t}^{-1}\right)}_{ll}}{a_{l}}\right)\\ \propto IG\left(\frac{\nu_{t}+L}{2},1/A_{l}^{2}+\nu_{t}{\left(\Sigma_{t}^{-1}\right)}_{ll}\right) \end{array}$$ (A.7)

with l=1,..,L and ${\left(\Sigma_{t}^{-1}\right)}_{ll}$ denotes the (l,l) entry of $\Sigma_{t}^{-1}$.

#### Full conditional for $\sigma_{Ei}^{2},$ with i=1,..,I.

$$\begin{array}{l} P\left(\sigma_{Ei}^{2}|\mathrm{ELSE}\right)\propto P\left(b_{2i}|\sigma_{Ei}^{2}\right)P\left(\sigma_{Ei}^{2}|a_{Ei}\right)\\ \propto {\left|\sigma_{Ei}^{2}⊗G_{1}\right|}^{-\frac{1}{2}}\exp\left(-\frac{1}{2\sigma_{Ei}^{2}}b_{2i}^{T}G_{1}^{-1}b_{2i}\right)P\left(\sigma_{Ei}^{2}|a_{Ei}\right)\\ \propto \sigma_{Ei}^{2}{}^{-\frac{\nu_{Ei}+1+JL-1+1+1}{2}}\exp\left(-\frac{1}{2}\left(\mathrm{tr}\{\left[b_{2i}^{T}G_{1}^{-1}b_{2i}+B_{Ei}\right]\sigma_{Ei}^{-2}\}\right)\right){\left|B_{Ei}\right|}^{\frac{\nu_{Ei}+1-1}{2}}\\ \propto IW\left(\kappa^{*}=\nu_{Ei}+1+JL-1,B_{E}^{*}=\left[b_{2i}^{T}G_{1}^{-1}b_{2i}+\frac{2\nu_{Ei}}{a_{Ei}}\right]\right) \end{array}$$ (A.8)

Since $B_{Ei}=\frac{2\nu_{Ei}}{a_{Ei}}$. With i=1,..,I and $b_{2i}={\left[b_{2i1}^{T},\ldots ,b_{2iJ}^{T}\right]}^{T}$.

#### Full conditional for $a_{Ei},$ with i=1,..,I.

$$\begin{array}{c} P\left(a_{Ei}\text{|}ELSE\right)\propto P(\sigma_{Ei}^{2}|a_{Ei})P(a_{Ei})\\ \propto \frac{1}{{\left(a_{Ei}\right)}^{\frac{\nu_{Ei}+1}{2}+1}}\exp\left(-\frac{1/A_{Ei}^{2}+\nu_{Ei}/\sigma_{Ei}^{2}}{a_{Ei}}\right)\\ \propto IG\left(\frac{\nu_{Ei}+1}{2},1/A_{Ei}^{2}+\nu_{Ei}/\sigma_{Ei}^{2}\right) \end{array}$$ (A.9)

#### Full conditional for *$R_{e}$* with l=1,…,L.

$$P\left(R_{e}\text{|}ELSE\right)\propto P(Y|\beta ,b_{1},b_{2},R_{e})P(R_{e}|a_{e1,}\ldots ,a_{eL}).$$

$$\begin{array}{l} \propto \frac{1}{{\left(R_{e}\right)}^{\frac{\nu_{e}+L+n-1+L+1}{2}}}\exp\left(-\frac{\mathrm{tr}\left\{\left[\sum_{i=1}^{I}\sum_{j=1}^{J}\sum_{k=1}^{K}e_{ijk}e_{ijk}^{T}+2\nu_{e}\text{diag}\left(\frac{1}{a_{e1}},\ldots ,\frac{1}{a_{eL}}\right)\right]R_{e}^{-1}\right\}}{2}\right){\left|B_{e}\right|}^{\frac{\nu_{e}+L-1}{2}}\\ \propto IW\left({\tilde{k}}_{bhl}=\nu_{e}+L+n-1,B_{e}^{*}=\sum_{i=1}^{I}\sum_{j=1}^{J}\sum_{k=1}^{K}e_{ijk}e_{ijk}^{T}+2\nu_{e}\text{diag}\left(\frac{1}{a_{e1}},\ldots ,\frac{1}{a_{eL}}\right)\right) \end{array}$$ (A.10)

with $B_{e}=2\nu_{e}\text{diag}\left(\frac{1}{a_{e1}},\ldots ,\frac{1}{a_{eL}}\right),$ $e_{ijk}=y_{ijk}-(X_{ijk}\beta +Z_{1ijk}b_{1j}+Z_{2ijk}b_{2ij})$, and l=1,2,3.

#### Full conditional for $a_{el}$

$$\begin{array}{l} P\left(a_{el}|\mathrm{ELSE}\right)\propto \text{P}\left(R_{e}|a_{el}\right)\text{P}\left(a_{el}\right)\\ \propto \frac{1}{{\left(a_{el}\right)}^{\frac{\nu_{e}+L}{2}+1}}\exp\left(-\frac{1/A_{el}^{2}+\nu_{e}{\left(R_{e}^{-1}\right)}_{ll}}{a_{el}}\right)\\ \propto IG\left(\frac{\nu_{e}+L}{2},1/A_{el}^{2}+\nu_{e}{\left(R_{e}^{-1}\right)}_{ll}\right) \end{array}$$ (A.11)

with l=1,2,3,…L and ${\left(R_{e}^{-1}\right)}_{ll}$ denotes the (l,l) entry of $R_{e}^{-1}$.

### Appendix B

#### Derivation of full conditional distributions for the BMTME diagonal model

All full conditional distributions of the BMTME diagonal model are the same as those of the BMTME unstructured model, except those needed for the variance–covariance ($\Sigma_{t},R_{e}),$ which are now diagonal. For this reason, here we provide the variances of the diagonal elements of $\Sigma_{t}$ ($\sigma_{t}^{2(l)},$ with l=1,…,L), $R_{e}$ ($\sigma_{e}^{2(l)},$ with l=1,…,L) and the required elements of a and $a_{e}$.

##### Full conditional for $\sigma_{t}^{2(l)}$

$P\left(\sigma_{t}^{2(l)}\text{|}ELSE\right)\propto IW(\kappa^{*}=\nu_{t}+J+1+JI-1,B_{t}^{*}=[{\left(b_{1}^{*}G_{g}^{-1}b_{1}^{*T}\right)}_{ll}+{\left(b_{2}^{*}G_{3}^{-1}b_{2}^{*T}\right)}_{ll}+2\nu_{t}(\frac{1}{a_{l}})$]) where ${\left(b_{1}^{*}G_{g}^{-1}b_{1}^{*T}\right)}_{ll}$ and ${\left(b_{2}^{*}G_{3}^{-1}b_{2}^{*T}\right)}_{ll}$ denote the (l,l) entry of the matrix $b_{1}^{*}G_{g}^{-1}b_{1}^{*T}$ and $b_{2}^{*}G_{3}^{-1}b_{2}^{*T}{}_{ll}$, respectively.

##### Full conditional for $a_{l}$

$$P\left(a_{l}\text{|}ELSE\right)\propto P(\sigma_{t}^{2(l)}|a_{l})P(a_{l})$$

$$\propto \frac{1}{{\left(a_{l}\right)}^{\frac{\nu_{t}+1}{2}+1}}\exp\left(-\frac{1/A_{l}^{2}+\nu_{t}\sigma_{t}^{-2(l)}}{a_{l}}\right)$$

$$\propto IG\left(\frac{\nu_{t}+1}{2},1/A_{l}^{2}+\nu_{t}/\sigma_{t}^{2(l)}\right)$$

##### Full conditional for $\sigma_{e}^{2(l)}$ with l=1,…,L

$$P\left(\sigma_{e}^{2\left(l\right)}|\mathrm{ELSE}\right)\propto IW\left({\tilde{k}}_{bhl}=\nu_{e}+1+n-1,B_{e}^{*}={\left(\sum_{i=1}^{I}\sum_{j=1}^{J}\sum_{k=1}^{K}e_{ijk}e_{ijk}^{T}\right)}_{ll}+\frac{2\nu_{e}}{a_{el}}\right)$$

where ${\left(\overset{I}{\underset{i=1}{\sum }}\overset{J}{\underset{j=1}{\sum }}\overset{K}{\underset{k=1}{\sum }}e_{ijk}e_{ijk}^{T}\right)}_{ll}$ denotes the (l,l) entry of the matrix $\overset{I}{\underset{i=1}{\sum }}\overset{J}{\underset{j=1}{\sum }}\overset{K}{\underset{k=1}{\sum }}e_{ijk}e_{ijk}^{T}$.

##### Full conditional for $a_{el}$

$$P\left(a_{el}\text{|}ELSE\right)\propto P(\sigma_{e}^{2(l)}|a_{el})P(a_{el})$$

$$\propto \frac{1}{{\left(a_{el}\right)}^{\frac{\nu_{e}+1}{2}+1}}\exp\left(-\frac{1/A_{el}^{2}+\nu_{e}\sigma_{e}^{-2(l)}}{a_{el}}\right)$$

$$\propto IG\left(\frac{\nu_{e}+1}{2},\frac{1}{A_{el}^{2}}+\frac{\nu_{e}}{\sigma_{e}^{2(l)}}\right)$$

### Appendix C

#### Derivation of full conditional distributions for the BMTME standard model

All full conditional distributions of the BMTME standard model are the same as those of the BMTME unstructured model, except those needed for the variance–covariance ($\Sigma_{t},\Sigma_{E},R_{e}),$ which are now equal to an identity multiplied by $\sigma_{t}^{2}$, $\sigma_{E}^{2}$, and $\sigma_{e}^{2}$, respectively. For this reason, here we provide the full conditional of $\sigma_{t}^{2}$, $\sigma_{E}^{2}$, and $\sigma_{e}^{2}$ and the required elements of $a_{t},$ $a_{E}$, and $a_{e}$.

##### Full conditional for $\sigma_{t}^{2}$

$$P\left(\sigma_{t}^{2}\text{|}ELSE\right)\propto IW(\kappa^{*}=\nu_{t}+JL+1+IJL-1,B_{t}^{*}=[b_{1}^{T}(G_{g}^{-1}⊗I_{L})b_{1}+b_{2}^{T}(G_{3}^{-1}⊗I_{L})b_{2}+2\nu_{t}(\frac{1}{a_{t}})])$$

$$\text{with}G_{3}=I_{I}\sigma_{E}^{2}⊗G_{g}.$$

##### Full conditional for $a_{t}$

$$P\left(a_{t}\text{|}ELSE\right)\propto P(\sigma_{t}^{2}|a_{t})\text{P}(a_{t})\propto \frac{1}{{\left(a_{t}\right)}^{\frac{\nu_{t}+1}{2}+1}}\exp\left(-\frac{1/A_{t}^{2}+\nu_{t}\sigma_{t}^{-2}}{a_{t}}\right)$$

$$\propto IG\left(\frac{\nu_{t}+1}{2},1/A_{t}^{2}+\nu_{t}/\sigma_{t}^{2}\right)$$

##### Full conditional for $\sigma_{E}^{2}$

$$P\left(\sigma_{E}^{2}\text{|}ELSE\right)\propto IW(\kappa^{*}=\nu_{E}+1+IJL-1,B_{t}^{*}=[b_{2}^{T}(I_{I}⊗G_{1}^{-1})b_{2}+2\nu_{E}(\frac{1}{a_{E}})])$$

$$\text{with}G_{1}=G_{g}⊗I_{L}\sigma_{t}^{2}.$$

##### Full conditional for $a_{E}$

$$P\left(a_{E}\text{|}ELSE\right)\propto P(\sigma_{E}^{2}|a_{E})\text{P}(a_{E})\propto \frac{1}{{\left(a_{E}\right)}^{\frac{\nu_{E}+1}{2}+1}}\exp\left(-\frac{1/A_{E}^{2}+\nu_{E}\sigma_{E}^{-2}}{a_{E}}\right)$$

$$\propto IG\left(\frac{\nu_{E}+1}{2},1/A_{E}^{2}+\nu_{E}/\sigma_{E}^{2}\right)$$

##### Full conditional for $\sigma_{e}^{2}$

$$P\left(\sigma_{e}^{2}\text{|}ELSE\right)\propto IW({\tilde{k}}_{bhl}=\nu_{e}+1+nL-1,B_{e}^{*}=e^{T}e+\frac{2\nu_{e}}{a_{e}})$$

where $e=Y-\mathrm{X\beta}-Z_{1}b_{1}-Z_{2}b_{2}$.

##### Full conditional for $a_{e}$

$$P\left(a_{e}\text{|}ELSE\right)\propto P(\sigma_{e}^{2}|a_{e})P(a_{e})$$

$$\propto \frac{1}{{\left(a_{e}\right)}^{\frac{\nu_{e}+1}{2}+1}}\exp\left(-\frac{1/A_{e}^{2}+\nu_{e}\sigma_{e}^{-2}}{a_{e}}\right)$$

$$\propto IG\left(\frac{\nu_{e}+1}{2},\frac{1}{A_{e}^{2}}+\frac{\nu_{e}}{\sigma_{e}^{2}}\right)$$

### Appendix D

**Table D1:** Cross-validation schemes

Line	Trait	CV1			CV2		
		env1	env2	env3	env1	env2	env3
1	1	y11 (1)	y21 (1)	y31 (1)	y11 (1)	y21 (1)	M
1	2	y11 (2)	y21 (2)	y31 (2)	y11 (2)	y21 (2)	y31 (2)
1	3	y11 (3)	y21 (3)	y31 (3)	y11 (3)	y21 (3)	y31 (3)
2	1	M	y22 (1)	y32 (1)	y12 (1)	y22 (1)	M
2	2	M	y22 (2)	y32 (2)	y12 (2)	y22 (2)	y32 (2)
2	3	M	y22 (3)	y32 (3)	y12 (3)	y22 (3)	y32 (3)
3	1	y13 (1)	y23 (1)	y33 (1)	y13 (1)	y23 (1)	M
3	2	y13 (2)	y23 (2)	y33 (2)	y13 (2)	y23 (2)	y33 (2)
3	3	y13 (3)	y23 (3)	y33 (3)	y13 (3)	y23 (3)	y33 (3)
4	1	y14 (1)	y24 (1)	y34 (1)	y14 (1)	y24 (1)	M
4	2	y14 (2)	y24 (2)	y34 (2)	y14 (2)	y24 (2)	y34 (2)
4	3	y14 (3)	y24 (3)	y34 (3)	y14 (3)	y24 (3)	y34 (3)
5	1	y15 (1)	y25 (1)	y35 (1)	y15 (1)	y25 (1)	M
5	2	y15 (2)	y25 (2)	y35 (2)	y15 (2)	y25 (2)	y35 (2)
5	3	y15 (3)	y25 (3)	y35 (3)	y15 (3)	y25 (3)	y35 (3)
6	1	y16 (1)	M	M	y16 (1)	y26 (1)	M
6	2	y16 (2)	M	M	y16 (2)	y26 (2)	y36 (2)
6	3	y16 (3)	M	M	y16 (3)	y26 (3)	y36 (3)
7	1	y17 (1)	y27 (1)	y37 (1)	y17 (1)	y27 (1)	M
7	2	y17 (2)	y27 (2)	y37 (2)	y17 (2)	y27 (2)	y37 (2)
7	3	y17 (3)	y27 (3)	y37 (3)	y17 (3)	y27 (3)	y37 (3)
8	1	y18 (1)	y28 (1)	y38 (1)	y18 (1)	y28 (1)	M
8	2	y18 (2)	y28 (2)	y38 (2)	y18 (2)	y28 (2)	y38 (2)
8	3	y18 (3)	y28 (3)	y38 (3)	y18 (3)	y28 (3)	y38 (3)
9	1	y19 (1)	y29 (1)	y39 (1)	y19 (1)	y29 (1)	M
9	2	y19 (2)	y29 (2)	y39 (2)	y19 (2)	y29 (2)	y39 (2)
9	3	y19 (3)	y29 (3)	y39 (3)	y19 (3)	y29 (3)	y39 (3)
10	1	M	M	y310 (1)	y110 (1)	y210 (1)	M
10	2	M	M	y310 (2)	y110 (2)	y210 (2)	y310 (2)
10	3	M	M	y310 (3)	y110 (3)	y210 (3)	y310 (3)
…	…	…	…	…	…	…	…
J-10	1	y1(J-10) (1)	y2(J-10) (1)	y3(J-10) (1)	y1(J-10) (1)	y2(J-10) (1)	M
J-10	2	y1(J-10) (2)	y2(J-10) (2)	y3(J-10) (2)	y1(J-10) (2)	y2(J-10) (2)	y3(J-10) (2)
J-10	3	y1(J-10) (3)	y2(J-10) (3)	y3(J-10) (3)	y1(J-10) (3)	y2(J-10) (3)	y3(J-10) (3)
J-9	1	y1(J-9) (1)	y2(J-9) (1)	y3(J-9) (1)	y1(J-9) (1)	y2(J-9) (1)	M
J-9	2	y1(J-9) (2)	y2(J-9) (2)	y3(J-9) (2)	y1(J-9) (2)	y2(J-9) (2)	y3(J-9) (2)
J-9	3	y1(J-9) (3)	y2(J-9) (3)	y3(J-9) (3)	y1(J-9) (3)	y2(J-9) (3)	y3(J-9) (3)
J-8	1	y1(J-8) (1)	M	y3(J-8) (1)	y1(J-8) (1)	y2(J-8) (1)	M
J-8	2	y1(J-8) (2)	M	y3(J-8) (2)	y1(J-8) (2)	y2(J-8) (2)	y3(J-8) (2)
J-8	3	y1(J-8) (3)	M	y3(J-8) (3)	y1(J-8) (3)	y2(J-8) (3)	y3(J-8) (3)
J-7	1	y1(J-7) (1)	y2(J-7) (1)	y3(J-7) (1)	y1(J-7) (1)	y2(J-7) (1)	M
J-7	2	y1(J-7) (2)	y2(J-7) (2)	y3(J-7) (2)	y1(J-7) (2)	y2(J-7) (2)	y3(J-7) (2)
J-7	3	y1(J-7) (3)	y2(J-7) (3)	y3(J-7) (3)	y1(J-7) (3)	y2(J-7) (3)	y3(J-7) (3)
J-6	1	y1(J-6) (1)	y2(J-6) (1)	y3(J-6) (1)	y1(J-6) (1)	y2(J-6) (1)	M
J-6	2	y1(J-6) (2)	y2(J-6) (2)	y3(J-6) (2)	y1(J-6) (2)	y2(J-6) (2)	y3(J-6) (2)
J-6	3	y1(J-6) (3)	y2(J-6) (3)	y3(J-6) (3)	y1(J-6) (3)	y2(J-6) (3)	y3(J-6) (3)
J-5	1	M	M	y3(J-5) (1)	y1(J-5) (1)	y2(J-5) (1)	M
J-5	2	M	M	y3(J-5) (2)	y1(J-5) (2)	y2(J-5) (2)	y3(J-5) (2)
J-5	3	M	M	y3(J-5) (3)	y1(J-5) (3)	y2(J-5) (3)	y3(J-5) (3)
J-4	1	y1(J-4) (1)	y2(J-4) (1)	y3(J-4) (1)	y1(J-4) (1)	y2(J-4) (1)	M
J-4	2	y1(J-4) (2)	y2(J-4) (2)	y3(J-4) (2)	y1(J-4) (2)	y2(J-4) (2)	y3(J-4) (2)
J-4	3	y1(J-4) (3)	y2(J-4) (3)	y3(J-4) (3)	y1(J-4) (3)	y2(J-4) (3)	y3(J-4) (3)
J-3	1	y1(J-3) (1)	y2(J-3) (1)	y3(J-3) (1)	y1(J-3) (1)	y2(J-3) (1)	M
J-3	2	y1(J-3) (2)	y2(J-3) (2)	y3(J-3) (2)	y1(J-3) (2)	y2(J-3) (2)	y3(J-3) (2)
J-3	3	y1(J-3) (3)	y2(J-3) (3)	y3(J-3) (3)	y1(J-3) (3)	y2(J-3) (3)	y3(J-3) (3)
J-2	1	y1(J-2) (1)	y2(J-2) (1)	M	y1(J-2) (1)	y2(J-2) (1)	M
J-2	2	y1(J-2) (2)	y2(J-2) (2)	M	y1(J-2) (2)	y2(J-2) (2)	y3(J-2) (2)
J-2	3	y1(J-2) (3)	y2(J-2) (3)	M	y1(J-2) (3)	y2(J-2) (3)	y3(J-2) (3)
J-1	1	y1(J-1) (1)	y2(J-1) (1)	y3(J-1) (1)	y1(J-1) (1)	y2(J-1) (1)	M
J-1	2	y1(J-1) (2)	y2(J-1) (2)	y3(J-1) (2)	y1(J-1) (2)	y2(J-1) (2)	y3(J-1) (2)
J-1	3	y1(J-1) (3)	y2(J-1) (3)	y3(J-1) (3)	y1(J-1) (3)	y2(J-1) (3)	y3(J-1) (3)
J	1	y1J (1)	y2J (1)	y3J (1)	y1J (1)	y2J (1)	M
J	2	y1J (2)	y2J (2)	y3J (2)	y1J (2)	y2J (2)	y3J (2)
J	3	y1J (3)	y2J (3)	y3J (3)	y1J (3)	y2J (3)	y3J (3)

In cross-validation 1 (CV1) lines were evaluated in some environments with all traits but are missing (M) in other environments (for all traits). Cross-validation 2 (CV2) simulates a situation where a trait is lacking in all lines in one environment but present in the remaining environments. Example of onefold cross-validation for J lines, three environments and three traits where the env are the environments. Yij(*l*) represents the response variable measured in environment i, genotype j, and trait *l*. For simplification we ignore the subscript of replication (k).

### Appendix E

Data preparation for analysis with I environments, J lines, K replications, and L traits is shown in the table below. gid denotes unique lines name, env are the environments, rep denotes the replications, and resp represents the response variables.

**ThreeWay:**

Trait	gid	env	rep	resp
1	G1	Env1	1	y111(1)
⋮	⋮	⋮	⋮	⋮
1	G1	Env1	K	y11K(1)
⋮	⋮	⋮	⋮	⋮
L	G1	Env1	K	y11K(L)
⋮	⋮	⋮	⋮	⋮
1	GJ	Env1	1	y1J1(1)
⋮	⋮	⋮	⋮	⋮
1	GJ	Env1	K	y1JK(1)
⋮	⋮	⋮	⋮	⋮
L	GJ	Env1	K	y1JK(L)
⋮	⋮	⋮	⋮	⋮
1	G1	EnvI	1	yI11(1)
⋮	⋮	⋮	⋮	⋮
1	G1	EnvI	K	yI1K(1)
⋮	⋮	⋮	⋮	⋮
L	G1	EnvI	K	yI1K(L)
⋮	⋮	⋮	⋮	⋮
1	GJ	EnvI	1	yIJ1(1)
⋮	⋮	⋮	⋮	⋮
1	GJ	EnvI	K	yIJK(1)
⋮	⋮	⋮	⋮	⋮
L	GJ	EnvI	K	yIJK(L)

### Appendix F

#### How to install and use the BMTME package

The BMTME package performs the proposed models (unstructured, diagonal, and standard).

Step 1. Install R-software version 3.2.4.

Step 2. Manually install the BMTME package that is available at the link: http://hdl.handle.net/11529/10646

Step 3. Use the package. Open R and copy and page Example 1.

################################ Example 1 #################################

rm(list = objects()); ls()

library(BMTME)

> data(ThreeWay) # load the built in package data file

> # to run with other data: load your data

## Transforming to the data to be used. Here you do not need to modify anything.

ThreeWay = transform(ThreeWay,

trait = factor(trait),

gid = factor(gid),

env = factor(env),

rep = factor(rep)

## Creating the GRM matrix for this example (here you need to upload your own genomic relationship matrix ###########################################################

K_x <- matrix(.7, ncol = 10, nrow = 10)

diag(K_x) <- 1

K <- diag(8) %x% K_x

ISigmaG <- solve(K)

####Here the model is fitted. You are only allowed to change model (“un” for unstructured #covariance matrix, “bd” for diagonal, and “st” for standard), nChain, nIter, and the working #directory (getwd ()) where you want to save your output. The output will be the β #coefficients, random effects b1 and b2, the three variance–covariances matrices (Sigma #Trait, Sigma Environments, and Sigma Residual of traits). The order of the call should be as follows considering the corresponding names in the dataframe ###########################

fit1<- fit(formula = resp ∼ trait + gid + env + rep,

data = ThreeWay,

K = ISigmaG,

model = ’un’,

nChain = 1,

nIter = 100,

saveAt = getwd(C:\\Osval\\))
